# Stable relocation of the radial head without annular ligament reconstruction using the Ilizarov technique to treat neglected Monteggia fracture: two case reports

**DOI:** 10.1186/1752-1947-4-344

**Published:** 2010-10-26

**Authors:** Altaf A Kawoosa, Shabir A Dhar, Mohammed Farooq Butt, Shareef A Wani, M R Mir, T A Dar

**Affiliations:** 1 Department of Orthopaedics, Government Medical College, Srinagar, Jammu and Kashmir, India

## Abstract

**Introduction:**

A Monteggia facture dislocation is not an uncommon injury, and the diagnosis can often be missed. Long-term follow-up of untreated Monteggia fracture dislocations reveals development of premature arthritis, pain, instability, and loss of pronation and supination. Methods involving annular ligament reconstruction require post-operative immobilization and use of transcapitellar pinning for maintenance of reduction, and thus a delay in rehabilitation. The literature reports satisfactory results with methods that involve ulnar osteotomy and open reduction of the radial head without annular ligament reconstruction. We used the Ilizarov method in two cases with neglected Monteggia fracture dislocations to stably reduce the radial head without open reduction and annular ligament reconstruction.

**Case presentation:**

We report two cases of neglected Monteggia fracture dislocation, in two Kashmiri boys aged four and six years. Using ulnar osteotomy with distraction osteogenesis, we were able to relocate the radial head gradually and maintain the reduction without a requirement for open reduction and annular ligament reconstruction.

**Conclusion:**

Distraction lengthening and hyperangulation in different planes by use of the Ilizarov technique effectively reduces the radial head without open reduction and annular ligament reconstruction.

## Introduction

Giovanni Battista Monteggia first described in 1814 the fracture dislocation now named after him. It represents a link between injuries of the forearm and the elbow [1]. These injuries follow the course of forearm fractures prognostically if recognized and treated early. However these injuries are often missed at the time of initial trauma [2,3].

Long-term follow-up of untreated Monteggia fracture dislocations reveals development of premature arthritis, pain, instability, and loss of pronation and supination [4,5]. Thus, it is imperative to treat the neglected fracture as soon as it is diagnosed. Freedman *et al. *performed reconstructive procedures up to six years after injury [6]. Currently, chronic dislocations are treated by ulnar osteotomy, open reduction of the radial head and reconstruction of the annular ligament [7]. The literature reports excellent results [8,9], but with restricted movement [10,11] and development of complications [12] associated with methods involving open reduction of the radial head, and ulnar ostoetomy with or without annular ligament reconstruction. Hirayama *et al.*, considering the interosseous membrane as the stabilizer of the radial head, described reduction of the radial head by hyperangulation and lengthening of the ulna without reconstruction of the annular ligament [13]. Relocation of the radial head has been successfully achieved by using distraction lengthening and hyperangulation over a uniplanar lengthening device [14].

To create hyperanglation in two planes, we used the Ilizarov method in two cases with neglected Monteggia fracture dislocations. Using ulnar osteotomy with distraction osteogenesis, we were able to relocate the radial head gradually. Our follow-up supports the view that distraction lengthening and hyperangulation in different planes may obviate the need for open reduction and annular ligament reconstruction.

## Case presentation

Our first patient was a four-year-old Kashmiri boy, who had pain and deformity of the right elbow. The child had received trauma to the elbow six months previously. On physical examination, a prominent radial head and mild limitation of supination and pronation were seen. Our second patient was a six-year-old Kashmiri boy. He had a history of elbow trauma one year previously, for which he had not received any treatment at the time.

**Figure 1:**
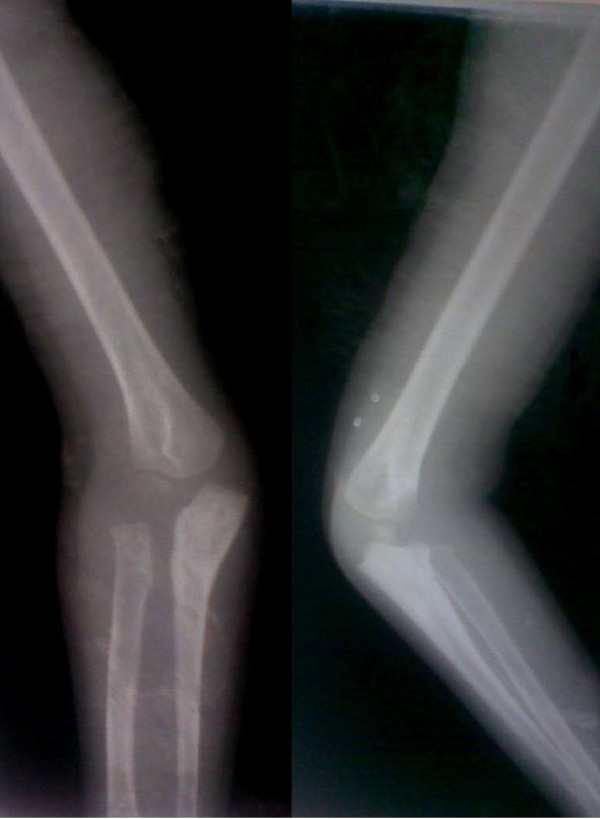
**Preoperative radiograph of patient 1 showing Monteggia fracture dislocation (Patient 1)**.

In both cases, an anterolateral dislocation of the radial head was confirmed by radiography. An underlying ulnar injury in both our cases was suspected because of the loss of proximal convexity of ulna (Figure 1).We chose a procedure (the Ilizarov technique) that would produce controlled lengthening and hyperangulation in two planes to restore the radiocapitellar articulation without open reduction and reconstruction of annular ligament. The procedure was explained to both sets of parents and written informed consent obtained from them. Approval from the institutional ethics board was also obtained.

**Figure 2:**
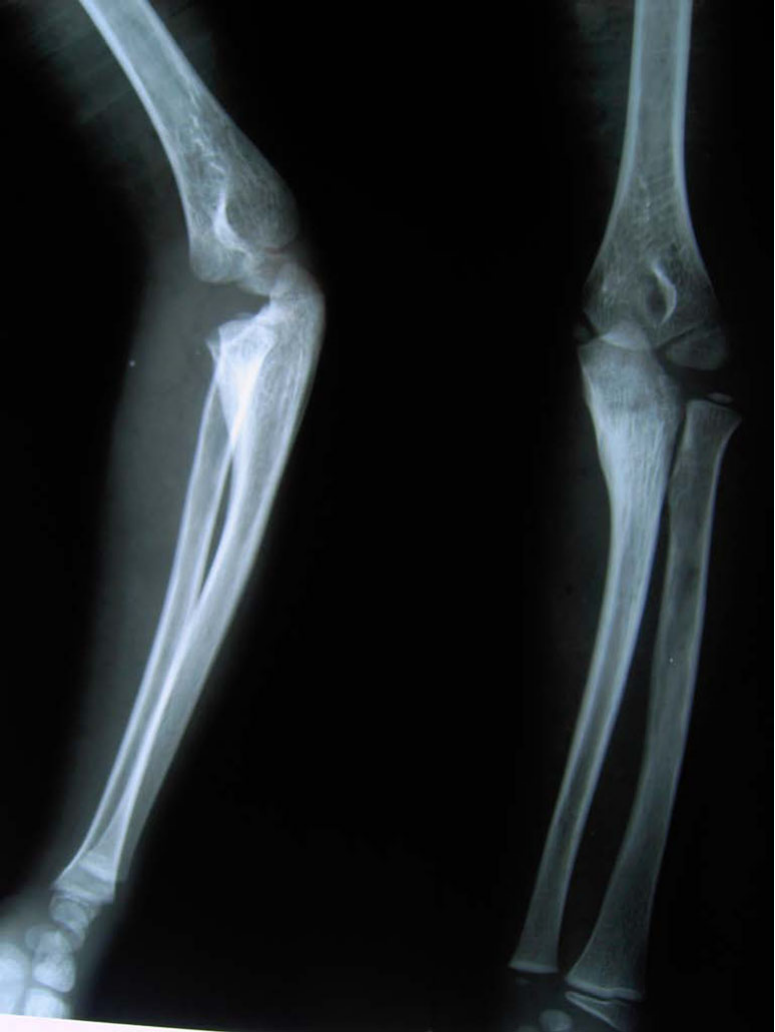
**Preoperative radiograph showing radial head dislocation (Patient 2)**.

**Figure 3:**
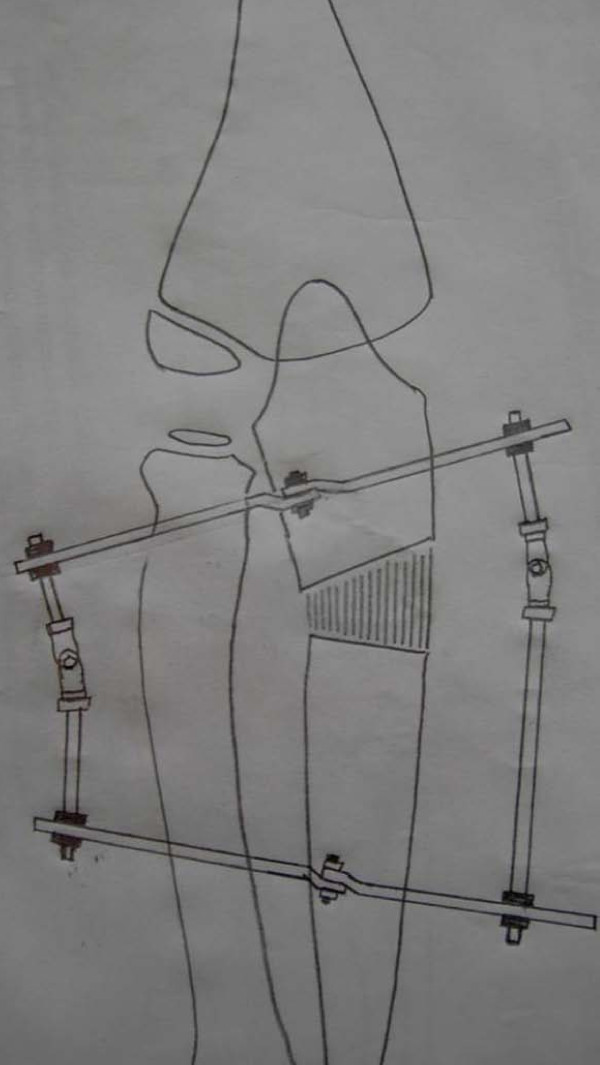
**Sketch showing planning for reduction of the radial head with hyperangulation of the regenerate in the anterior-posterior planes**.

**Figure 4:**
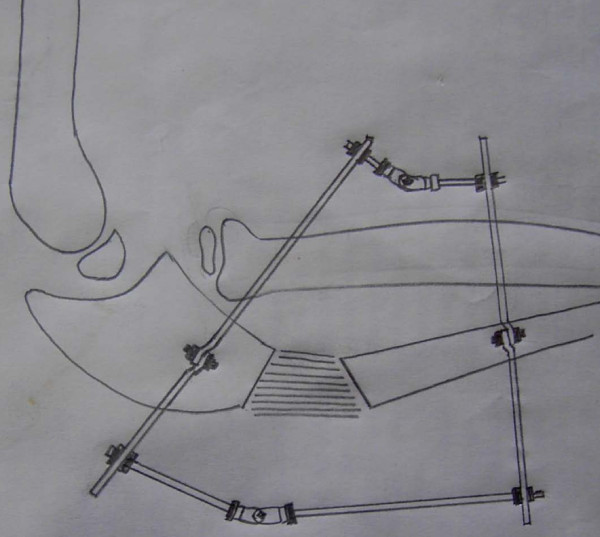
**Sketch showing planning for reduction of the radial head with hyperangulation of the regenerate in lateral planes**.

Radiographs in both anterio-posterior and lateral view were studied to assess the dislocation of the radial head. Because the dislocation in both of our patients was an anterolateral one (Figures 1, 2), an osteotomy in the proximal ulna and differential lengthening in two planes was planned to create a medial (Figure 3) and posterior (Figure 4) hyperangulation, to place the radial head in the appropriate radiocapitellar orientation. We hoped to avoid open reduction of the dislocation and reconstruction of the annular ligament.

**Figure 5:**
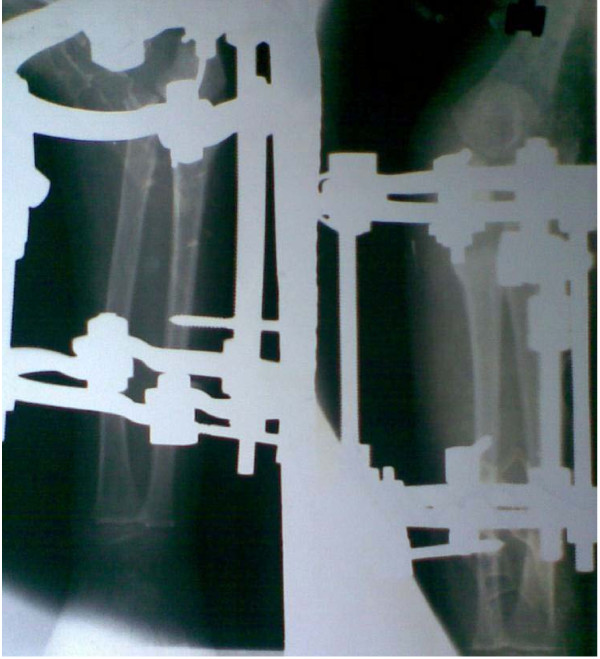
**Postoperative radiograph after application of Ilizarov fixator and ulnar osteotomy (Patient 1)**.

The surgery was undertaken under general anesthesia. A two-ring construct with hinge application was used. The ring was fixed only to the ulna, to allow free supination and pronation movement. The proximal ring was fixed with an Ilizarov wire and one half-pin. The distal ring was fixed by two half-pins in different planes on the subcutaneous border. Through an incision 15 mm long, a low-energy corticotomy of the ulna was performed at the proposed site (Figure 5). We did not make any attempt to hyperangulate the ostotomy intraoperatively. Distraction was started on the seventh day after surgery in a differential manner, to create lengthening and hyperangulation in two planes as planned.

**Figure 6:**
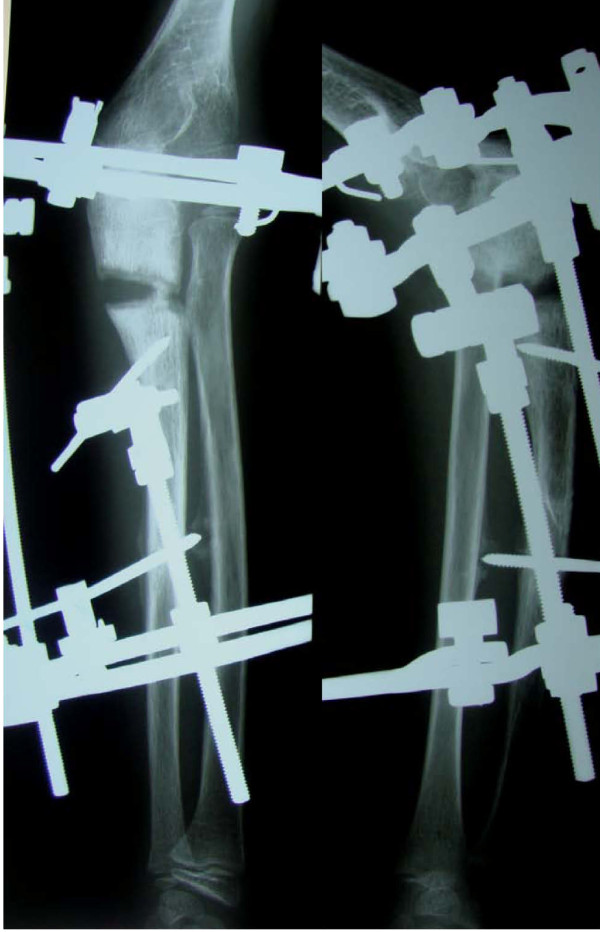
**Postoperative radiograph during disraction lengthening (Patient 2)**.

**Figure 7:**
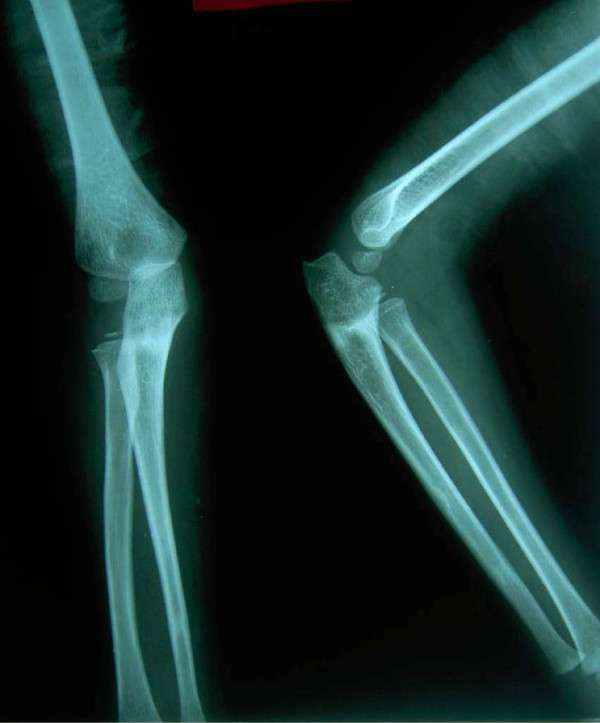
**Radiograph taken at the two-year follow-up visit showng excellent relocation of the radial head (Patient 1)**.

**Figure 8:**
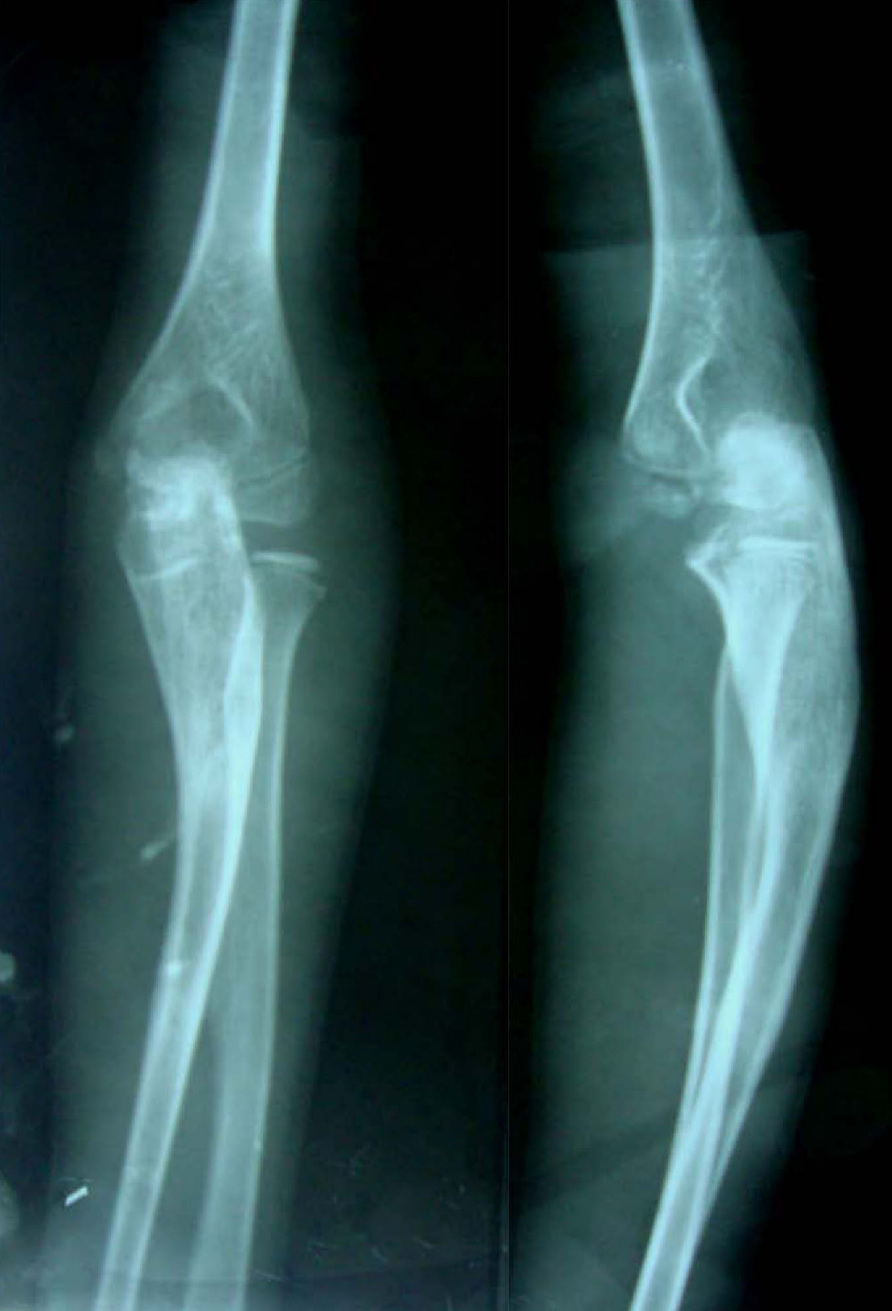
**Radiograph taken at the final follow-up visit showing relocation of the radial head (Patient 2)**.

We followed up the progress of our patients every week with both clinical and radiologic examinations to assess the lengthening, angulation and relocation of the radial head. For our first patient, relocation of the radial head was confirmed both clinically and by radiography by the third postoperative week. Relocation took longer for our second patient, being achieved by the fifth postoperative week and involving lengthening of the ulna by 15 mm (Figure 6). Both patients were encouraged to perform range of motion exercises of the elbow, and the frame was left in place until maturation of the regenerated bone. The ring was finally taken off at six weeks for our first patient and at 12 weeks for our second patient; for both, a protective long arm cast was applied for another two weeks. The regenerated bone healed at an average rate of three weeks/cm. The radial head maintained the reduced position without annular ligament reconstruction (Figures 7, 8).

**Figure 9:**
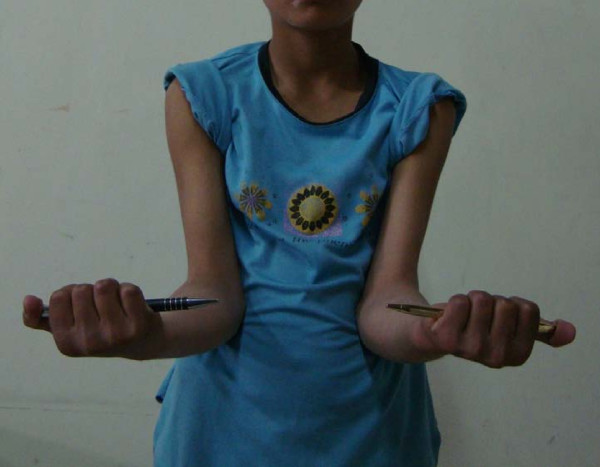
**Supination at elbow**.

**Figure 10:**
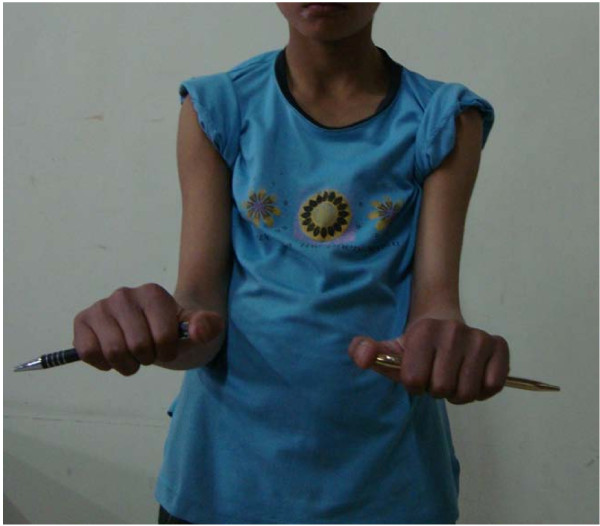
**Pronation at elbow**.

At follow-up two years after surgery, both patients had an excellent result and 100% range of motion around the affected elbow (Figures 9, 10).

## Discussion

Owing to the potential complications of conservative methods in cases of neglected Monteggia injuries, it is important that the radial head be replaced in appropriate relation to the capitellum. This is especially true in children who are less than 12 years of age.

In procedures involving reconstruction around the radial head, it is mandatory to stabilize the radial head by a transcapitellar K wire to prevent redislocation [4,7]. In one study, Hori *et al. *reported that of the 13 patients treated surgically with open reduction, ulnar osteotomy and annular ligament reconstruction, there were seven re-dislocations, and seven patients had restriction of movement [15]. The authors reported that they achieved better results once they modified the osteotomy procedure, and stressed the need for angulation and elongation of the osteotomy. Rodgers *et al. *reported 14 complications in seven patients, including problems with the osteotomy site, re-dislocations and nerve injuries [12].

Restriction of movement has been seen frequently in procedures involving radial head reconstruction [10,11]. Problems with the fixation of the osteotomy are not uncommon [13]. Hasler found it easy to reconstruct the radiocapitellar joint, and to allow early resumption of functional exercises by using external fixation for the ulnar osteotomy with no annular ligament reconstruction [16]. Freedman *et al. *used a technique in which the annular ligament was not reconstructed but the radial notch was deepened to achieve stability [6].

Chronic dislocations may be associated with significant discrepancy in the radioulnar length, the radius having overgrown as a result of lack of support between the capitellum and the radial head. The optimum treatment in these patients is lengthening of the osteotomy in addition to angulation. The excellent results of a lengthening osteotomy are best reflected in the cases presented by Exner, who used distraction lengthening over a uniplanar lengthening device to create hyperangulation and lengthening to reduce the radial head [14]. However, the process of hyperangulation was performed after distraction at subsequent stages under analgesia. We found the Ilizarov technique to be advantageous in other respects as well, as it allows lengthening and angulation in several planes, and the processes of lengthening and angulation proceed simultaneously.

The advantages of distraction treatment can be summarized as: minimally invasive surgery, controlled lengthening, no radiocapitellar intervention, no need for bone grafting, and early resumption of functional exercises. The only difficulty encountered during the process of correction in our patients was the radiologic interpretation of the relocation of the radial head, because of the superimposing proximal steel ring; however, this problem can be easily managed by the use of carbon fiber radiolucent rings.

## Conclusion

Use of ulnar osteotomy with distraction lengthening and hyperangulation in several planes with the Ilizarov technique could be an effective method of treating neglected Monteggia fracture dislocations.

## Competing interests

All authors confirm that there are no conflicts of interests, including financial and personal relationships with other people, or organizations, that could inappropriately influence (bias) their work.

## Consent

Written informed consent was obtained from the parents of both the patients for publication of this case report and accompanying images. A copy of the written consent is available for the review by the Editor in Chief of this journal.

## Authors' contributions

AAK was responsible for the concept, planning, surgery and manuscript preparation. The rest of the authors contributed in preparation of the manuscript. All authors have read and approved the final manuscript.

## References

[B1] Monteggia GB Instituzioni Chirurgiche 1814 Milan, Maspero

[B2] Dormans JP Rang M The problem of Monteggia fracture dislocation in children Orthop Clin North Am 1990 21 251 256 2326051

[B3] Fowles JV Sliman N Kassab MT Monteggia lesion in children: fracture of the ulna and dislocation of the radial head J Bone Joint Surg Am 1983 65 1276 1282 6654941

[B4] Bell-Tawse AJS The treatment of malunited anterior monteggia fractures in children JBJS 1965 47B 718 723 5846773

[B5] Kalamchi A Monteggia fracture dislocation in children JBJS 1986 68-A 615 619 3957989

[B6] Freedman L Luk K Leong JC Radial head reduction after missed monteggia fracture; Brief report JBJS 1988 70;B 846 847 10.1302/0301-620X.70B5.3192599 3192599

[B7] LIoyd Roberts GC Bucknill TM Anterior dislocation of the radial head in children: Aetiology, natural history and management J Bone Joint Surg (Br) 1977 58-B 402 407 10.1302/0301-620X.59B4.925049 925049

[B8] Degreef I Desmet L Missed radial head dislocationin children associated with ulnar deformity; treatment by open reduction and ulnar osteotomy J Orthop Trauma 2004 18 375 378 10.1097/00005131-200407000-00008 15213503

[B9] Wang Mn Chang Wn Chronic post traumatic anterior dislocation of the radial head in children; thirteen cases treated by open reduction, ulnar osteotomy and annular ligament reconstruction through Boyd incision J Orthop Trauma 2006 20 1 5 10.1097/01.bot.0000189881.75421.92 16424802

[B10] Devnani AS Missed Monteggia fracture dislocation in children Injury 28 131 133 10.1016/S0020-1383(96)00160-X 9205580

[B11] Stoll TM Willis RB Paterson DC Treatment of missed Monteggia fracture in the child J Bone Joint Surg Br 1992 74 436 440 1587897 10.1302/0301-620X.74B3.1587897

[B12] Rodgers WB Waters PM Hall JE Chronic Monteggia lesions in children: complications and results of reconstruction J Bone Joint Surg Am 1996 78 1322 1329 8816646 10.2106/00004623-199609000-00005

[B13] Hirayama T Takimitsu Y Yagihara K Mikita A Operation for chronic dislocation of the radial head in children; reduction by osteotomy of the ulna J Bone joint Sur (Br) 1987 69-B 639 642 10.1302/0301-620X.69B4.3611173 3611173

[B14] Exner GU Missed chronic anterior Monteggia lesion; Closed reduction by gradual lengthening and angulation of the ulna J Bone Joint Surg (BR) 2001 83-B 547 550 10.1302/0301-620X.83B4.11103 11380129

[B15] Horii E Nakamura R Koh S Inagaki H Yajima H Nakao E Surgical treatment for chronic radial head dislocation J Bone joint Surg Am 2002 84-A 1183 1188 12107319 10.2106/00004623-200207000-00014

[B16] Hasler CC Von Laer L Hell AK Open reduction, ulnar ostoetomy and external fixation for chronic anterior dislocation of the head of radius J Bone Joint Surg [Br] 2005 78 88 94 15686243

